# Comparative Analysis of Seven Viral Nuclear Export Signals (NESs) Reveals the Crucial Role of Nuclear Export Mediated by the Third NES Consensus Sequence of Nucleoprotein (NP) in Influenza A Virus Replication

**DOI:** 10.1371/journal.pone.0105081

**Published:** 2014-08-13

**Authors:** Nopporn Chutiwitoonchai, Michinori Kakisaka, Kazunori Yamada, Yoko Aida

**Affiliations:** 1 Viral Infectious Diseases Unit, RIKEN, Wako, Saitama, Japan; 2 Computational Biology Research Center, National Institute of Advanced Industrial Science and Technology, Koto, Tokyo, Japan; University of Rochester Medical Center, United States of America

## Abstract

The assembly of influenza virus progeny virions requires machinery that exports viral genomic ribonucleoproteins from the cell nucleus. Currently, seven nuclear export signal (NES) consensus sequences have been identified in different viral proteins, including NS1, NS2, M1, and NP. The present study examined the roles of viral NES consensus sequences and their significance in terms of viral replication and nuclear export. Mutation of the NP-NES3 consensus sequence resulted in a failure to rescue viruses using a reverse genetics approach, whereas mutation of the NS2-NES1 and NS2-NES2 sequences led to a strong reduction in viral replication kinetics compared with the wild-type sequence. While the viral replication kinetics for other NES mutant viruses were also lower than those of the wild-type, the difference was not so marked. Immunofluorescence analysis after transient expression of NP-NES3, NS2-NES1, or NS2-NES2 proteins in host cells showed that they accumulated in the cell nucleus. These results suggest that the NP-NES3 consensus sequence is mostly required for viral replication. Therefore, each of the hydrophobic (Φ) residues within this NES consensus sequence (Φ1, Φ2, Φ3, or Φ4) was mutated, and its viral replication and nuclear export function were analyzed. No viruses harboring NP-NES3 Φ2 or Φ3 mutants could be rescued. Consistent with this, the NP-NES3 Φ2 and Φ3 mutants showed reduced binding affinity with CRM1 in a pull-down assay, and both accumulated in the cell nucleus. Indeed, a nuclear export assay revealed that these mutant proteins showed lower nuclear export activity than the wild-type protein. Moreover, the Φ2 and Φ3 residues (along with other Φ residues) within the NP-NES3 consensus were highly conserved among different influenza A viruses, including human, avian, and swine. Taken together, these results suggest that the Φ2 and Φ3 residues within the NP-NES3 protein are important for its nuclear export function during viral replication.

## Introduction

Transmission and infection of seasonal influenza A viruses results in many human deaths every year and is a serious world-wide public health concern [1]. Influenza A subtypes H5N1, H7N7, and, more recently, H7N9 are potentially fatal viruses that have either re-emerged or newly emerged through avian-to-human transmission [1]–[3]. Influenza A virus belongs to the family *Orthomyxoviridae*, which comprises eight negative-sense single-stranded RNA genomes that encode polymerase acidic protein (PA), two polymerase basic proteins (PB1 and PB2), nucleoprotein (NP), matrix protein (M1), proton channel protein (M2), two non-structural proteins (NS1 and NS2), hemagglutinin (HA), and neuraminidase (NA) [4]. After the influenza virus enters human cells, the nuclear localization signal (NLS) on NP is recognized by cellular importin-α, and the viral ribonucleoproteins (vRNPs; the viral genome complexed with NP, PA, PB1, and PB2) are transported to the nucleus for viral mRNA synthesis and protein expression [5], [6]. To assemble progeny virions, the virus requires nuclear export machinery to transport the viral ribonucleoproteins (vRNP) complex to cytoplasm before trafficking to plasma membrane where the assembly process takes place [4], [7].

Export of macromolecules through the nuclear envelope requires that a unique leucine-rich consensus sequence, Φ1 X_2–3_Φ2 X_2–3_Φ3XΦ4 [where Φ represents hydrophobic residues (e.g., leucine, isoleucine, valine, or methionine) and X represents any amino acid], known as the nuclear export signal (NES) [8], is recognized by the specific cellular transport receptor, chromosome region maintenance 1 (CRM1) [9]. Human immunodeficiency virus type-1 (HIV-1) Rev and human cellular protein kinase inhibitor (PKI) are well-characterized NES-containing proteins involved in the nuclear export of unspliced viral RNA [10] and cellular protein kinase A [11], respectively. Influenza A virus NS2 (NS2-NES1; L12–L21) was initially thought to mediate the nuclear export of vRNPs; thus it was renamed “nuclear export protein” (NEP) [12]. The NS2 protein forms a complex with vRNP by interacting with the M1 protein; the vRNP is then exported to the cytoplasm via a CRM1-dependent pathway [12]–[14]. Later, a second NES within the NS2 protein was identified (NS2-NES2; M31–L40), which is located in the N2 helix region [15]. NS2-NES2 and NS2-NES1 coordinate and regulate the NS2-CRM1 interaction; however, deletion of NES1 actually increases NS2-CRM1 binding [15]. Moreover, a recent review shows that NS2 also plays a role in regulating viral RNA transcription and replication, which is thought to be involved in the adaptation of avian H5N1 to mammalian hosts [16]. The inactive form of NES (latent NES) resides at the end of the NS1 protein effector domain (NS1-NES; F138–L146) [17]. The NS1 protein harboring a latent NES localizes to the nucleus where it inhibits cellular pre-mRNA splicing and nuclear export of mRNA [17]. After the latent NES is unmasked (via a specific interaction with an as-yet-unidentified protein), NS1 is able to translocate to the cytoplasm; however, the significance of this event in terms of viral replication remains unclear [17], [18]. Recently, the NES of the M1 protein (M1-NES; I59–V68) was identified by NES motif analysis [19]. An NES-defective M1 protein was retained in the cell nucleus, thereby reducing both nuclear export of the vRNP and viral production efficiency [19]; however, the nuclear export function of M1-NES was not inhibited by leptomycin B (LMB), indicating that it did not rely on the CRM1-independent export pathway [19]. Interestingly, a recent study identified three NESs (NP-NES1; E24–L49, NP-NES2; V183–I197, and NP-NES3; P248–S274) within a single viral NP [20]. The nuclear export of NP-NES3 was CRM1-dependent, whereas that of NP-NES1 and NP-NES2 was CRM1-independent [20]. In addition, mutation of all three NESs resulted in the nuclear accumulation of NP and impaired viral production [20].

To identify the NES that is most suitable as a target for antiviral drug development and to better understand the role of nuclear export in viral replication, we constructed seven NES mutants from four identical viral proteins and compared their functions with respect to viral replication and nuclear export. Mutation of NS2-NES1 and NS2-NES2 reduced viral replication kinetics, whereas mutation of NP-NES3 meant that no viruses could be rescued using reverse genetics approaches. Consistent with this, these NES mutants were retained within the host cell nucleus. A single-point mutation at each Φ residue within the NP-NES3 consensus sequence indicated that Φ2 and Φ3 were critical for NP function involving CRM1 binding, nuclear export, and viral production.

## Results

### Among seven influenza virus NESs, NP-NES3 is the most critical for viral production and replication

Seven NESs that mediate the nuclear export of influenza viral proteins have been identified [12], [15], [17], [19], [20]. Therefore, to compare the roles of their NES consensus sequences in viral replication, we first constructed NES mutant plasmids as shown in Table 1. Since it was proposed that the last two Φ residues (Φ3 and Φ4) within the NES consensus sequence are critical for NES function [8], we replaced these Φ residues with alanine. The construction of four NES mutants; NS1-NES, NS2-NES1, NS2-NES2, and NP-NES3 were different from that in a previous report while the other three NES mutants; M1-NES, NP-NES1, and NP-NES2 were similar [12], [15], [17], [19], [20]. We used the NP-NES3 mutant harboring mutations in the Φ3 and Φ4 residues, which is different from a previous report in which a mutation was introduced into the Φ4 residue only [20]. Since the translational reading frame of NS2-NES partially overlaps with the NS1 sequence, we mutated the Φ residues within NS2-NES1 and NS2-NES2 so as to avoid any codon changes in the NS1 protein.

**Table 1:** List of NES consensus sequence mutants.

NES			Sequence^a^
Consensus			Φ1 X_2–3_ Φ2 X_2–3_ Φ3 X Φ4
NS1	NES	WT	^138^FDRLETLIL ^146^
		MT	- - - - - - A-A
NS2	NES1	WT	^12^ILMRMSKMQL ^21^
		MT	- - - - - - - -T-S
	NES2	WT	^31^ MITQFESLKL ^40^
		MT	- - - - - - - A-H
NP	NES1	WT	^32^ MIDGIGRFYI ^41^
		MT	- - - - - - - A-A
	NES2	WT	^183^ VKGVGTMVM ^191^
		MT	- - - - - - - A-A
	NES3	WT	^256^ LIFLARSALIL ^266^
		MT	- - - - - - - -A-A
M1	NES	WT	^59^ ILGFVFTLTV ^68^
		MT	- - - - - - -A-A

a Hydrophobic (Φ) residues, e.g., leucine, isoleucine, valine, and methionine, in the NES consensus sequence are underlined.

**Figure 1:**
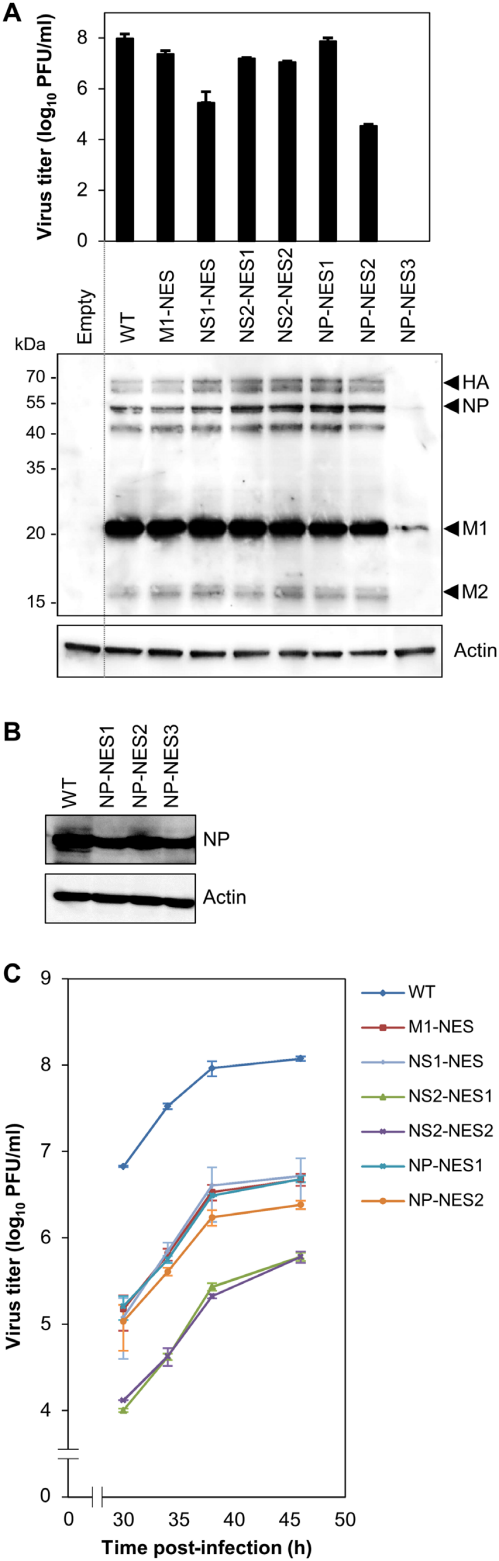
Production, protein expression, and replication kinetics of NES mutant viruses. (A) Wild-type NES or mutant viruses (M1-NES, NS1-NES, NS2-NES1, NS2-NES2, NP-NES1, NP-NES2, and NP-NES3) were produced via reverse genetics by co-culturing HEK-293T and MDCK cells transfected with eight viral genomic plasmids (pHH21 containing PA, PB1, PB2, HA, NA, NP, M, and NS) and four viral protein expression plasmids (pCAGGS containing PA, PB1, PB2, and NP) for 72 h. Supernatants containing viruses were collected, clarified, and titrated in a plaque assay on MDCK cells. Viral titration was performed in triplicate, and the plaque forming units PFU/ml (mean±SD) was calculated and plotted (upper panel). Viral protein expression in the producer cells was compared by collecting the cells, lysing them, and separating the proteins on 10% SDS-PAGE gels. Proteins were then blotted with either an anti-WSN antibody (Ab) or an anti-actin monoclonal antibody (MAb) (lower panel). The HA, NP, M1, and M2 proteins are shown alongside molecular weight markers. (B) Expression of NP-NES wild-type and mutant proteins was compared by transfecting NP/pHH21, NP/pCAGGS, PA/pCAGGS, PB1/pCAGGS, or PB2/pCAGGS into HEK-293T cells for 48 h. The cells were then lysed and blotted with either an anti-WSN Ab or an anti-actin MAb. (C) MDCK cells were infected with equal amounts of each of the viruses (MOI = 0.001) described in (A) and then cultured. At the indicated time-points post-infection, the viruses were collected and titrated in a plaque assay. Data are expressed as the mean (±SD) PFU/ml from triplicate titrations.

Infectious viruses were generated by reverse genetics using co-cultures of HEK-293T and Madin-Darby canine kidney (MDCK) cells transfected with eight viral genomic plasmids (pHH21; harboring PA, PB1, PB2, HA, NA, NP, M, and NS) and four viral protein expression plasmids (pCAGGS; harboring PA, PB1, PB2, and NP) [5], [21]. Plaque titration of supernatants containing the viruses revealed slightly lower titers for all NES mutant viruses than for the wild-type virus; in particular, no virus was detected in the supernatant from cells transfected with the NP-NES3 mutant (harboring substitutions L264A and L266A) (Fig. 1A, upper panel). The expression of viral proteins (i.e., HA, NP, NA, M1, and M2) by each NES mutant did not correlate with the virus titer, and was similar to that of the wild-type (with the exception of the NP-NES3 mutant) (Fig. 1A, lower panel). Although viral protein expression of the NP-NES3 mutant in producer cells was very low (Fig. 1A, lower panel), expression of the NP-NES3 mutant protein in HEK-293T cells transfected with NP-NES3/pHH21 plus PA/pCAGGS, PB1/pCAGGS, PB2/pCAGGS, and NP-NES3/pCAGGS was similar to that of the wild-type and the NP-NES1 and NP-NES2 mutants (Fig. 1B). This result indicates that mutation of the NES3 consensus sequence within NP had a marked effect on viral production.

We next compared the replication kinetics of the NES mutant viruses by infecting MDCK cells at a multiplicity of infection (MOI) of 0.001 and measuring virus titers at the indicated time-points. All NES mutant viruses showed lower levels of replication than the wild-type virus (10^2^-fold lower viral titer) during the exponential phase; however, the NS2-NES1 mutant (harboring substitutions at M19T and L21S) and the NS2-NES2 mutant (harboring substitutions at L38A and L40H) replicated particularly poorly (10^3^-fold lower titer than that of the wild-type) (Fig. 1C). The observed reduction in the replication kinetics of the M1-NES mutant was similar to that reported previously [19]; however, the lower replication kinetics of the other NES mutants is a novel finding (the titer of NS2-NES2 L38A/L40H was 10-fold lower than that in a previous report examining a mutant, NS2-NES2 L38A/L40A [15]).

**Figure 2:**
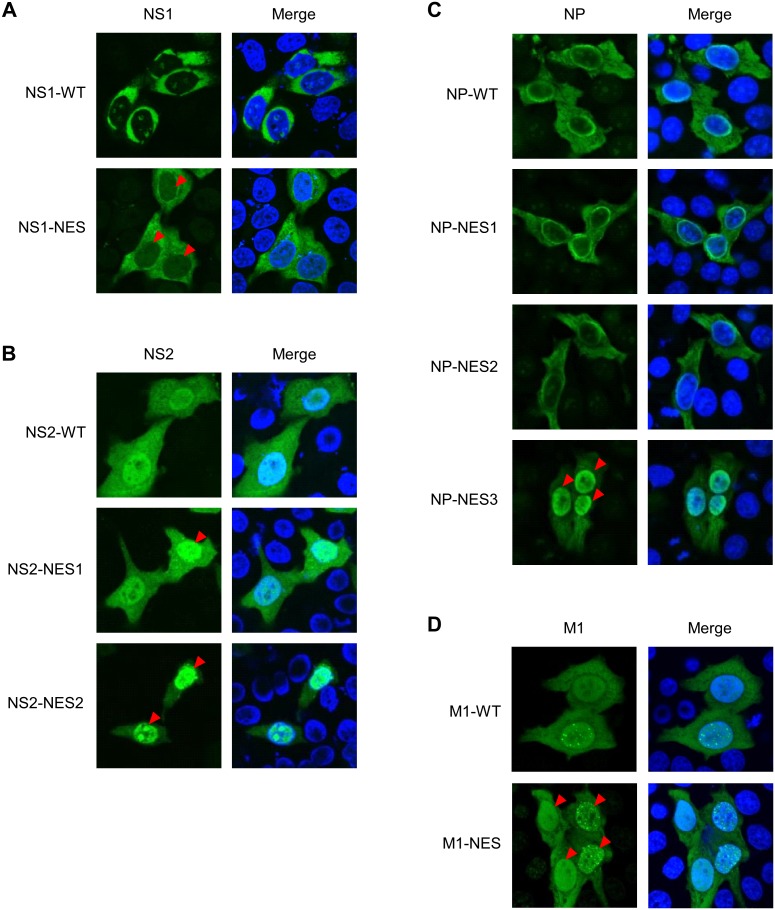
Intracellular localization of NES mutant proteins. HeLa cells were grown on cover glass and transfected with wild-type NS1 or NS1-NES mutant plasmid (A), wild-type NS2 or NS2-NES mutant (NS2-NES1 or NS2-NES2) plasmid (B), wild-type NP or NP-NES mutant (NP-NES1, NP-NES2, or NP-NES3) plasmid (C), or wild-type M1 or M1-NES mutant plasmid (D) for 48 h before immunofluorescence staining with anti-NS1 MAb, anti-NS2 Ab, anti-NP MAb, or anti-M1 MAb (respectively) followed by an Alexa Fluor 488-conjugated secondary antibody and Hoechst 333342. The cells were then observed under a confocal laser-scanning microscope. The red arrow heads indicate the localization change of NES mutant proteins (compare with wild-type proteins).

Since defective replication of NES mutant viruses appears to correlate with the nuclear export of viral proteins [15], [19], [20], we next examined the intracellular localization of these seven NES mutant proteins in HeLa cells transfected with PA/pCAGGS, PB1/pCAGGS, PB2/pCAGGS, and NP/pCAGGS together with either NS1/pHH21, NS2/pHH21, M1/pHH21, NS1-NES mutant/pHH21, NS2-NES mutant/pHH21, or M1-NES mutant/pHH21, or in the cells transfected with PA/pCAGGS, PB1/pCAGGS, PB2/pCAGGS, and NP/pCAGGS, or NP-NES mutant/pCAGGS together with either NP/pHH21 or NP-NES mutant/pHH21, by immunofluorescence staining. Different from previous reports [15], [17], [19], [20], all the NES mutant proteins examined in this study were untagged and full-length. The localization change of the NES mutant proteins is indicated by red arrow heads in Figure 2. NS1 wild-type mainly localized in the cytoplasm, whereas the NS1-NES mutant was slightly retained in the nucleus (Fig. 2A). By contrast, wild-type NS2 proteins were mainly observed in the nucleus (with some faint staining in the cytoplasm), whereas the NS2-NES1 mutant (partially) and NS2-NES2 mutant (mainly) proteins were trapped in the nucleus (Fig. 2B). The nuclear accumulation of the NS2-NES2 mutant protein (L38A/L40H) observed herein was different from that reported in a previous study showing that a green fluorescent protein (EGFP)-NS2-NES2 mutant (L38A/L40A) localized to the cytoplasm [15]. The wild-type, NP-NES1, and NP-NES2 mutant proteins mainly localized in the cytoplasm, whereas the NP-NES3 mutant was retained in the nucleus (Fig. 2C). These localization patterns of NP-NES mutant proteins were similar to those reported in a previous study (except for NP-NES3: the previous report showed that the localization of NP-NES3 was the same as that of the wild-type NP [20]). However, the NP-NES3 mutant used in the present study (L264A/L266A) is different from that used in the previous study (L266A) [20]. The M1-NES mutant protein showed a little high levels of accumulation in the nucleus when compared with the wild-type (Fig. 2D), which was similar to that reported previously for EGFP-M1 NES mutant [19].

As summarized in Table 2, nuclear accumulation of viral proteins correlated with a failure to rescue the NP-NES3 mutant using reverse genetics and with the slow replication kinetics of other NES mutants. Taken together, these data suggest that the nuclear export function of these proteins is important for viral production and replication.

**Table 2:** Summary of viral replication kinetics and nuclear localization of NES consensus sequence mutants.

Virus		Replication kinetics (%WT)^a^	Localization change^c^
NS1	WT	100.0±5.8	
	NES	4.4±2.6	±
NS2	WT	100.0±5.8	
	NES1	0.5±0.1	±
	NES2	0.5±0.1	+
NP	WT	100.0±5.8	
	NES1	4.1±0.4	−
	NES2	2.0±0.2	−
	NES3	-b	+
M1	WT	100.0±5.8	
	NES	4.0±0.6	±

a % virus titer ± SD compared with WT at the 46 h of replication kinetic assay from the Fig. 1C.

b No viral rescue by reverse genetics.

c –, ±, + indicate no change, partial change, great change of NES mutant protein localization compared with WT from the Fig. 2.

### NP-NES3 Φ1, Φ2, Φ3, and Φ4 are essential for viral production, replication, and nuclear export

**Figure 3:**
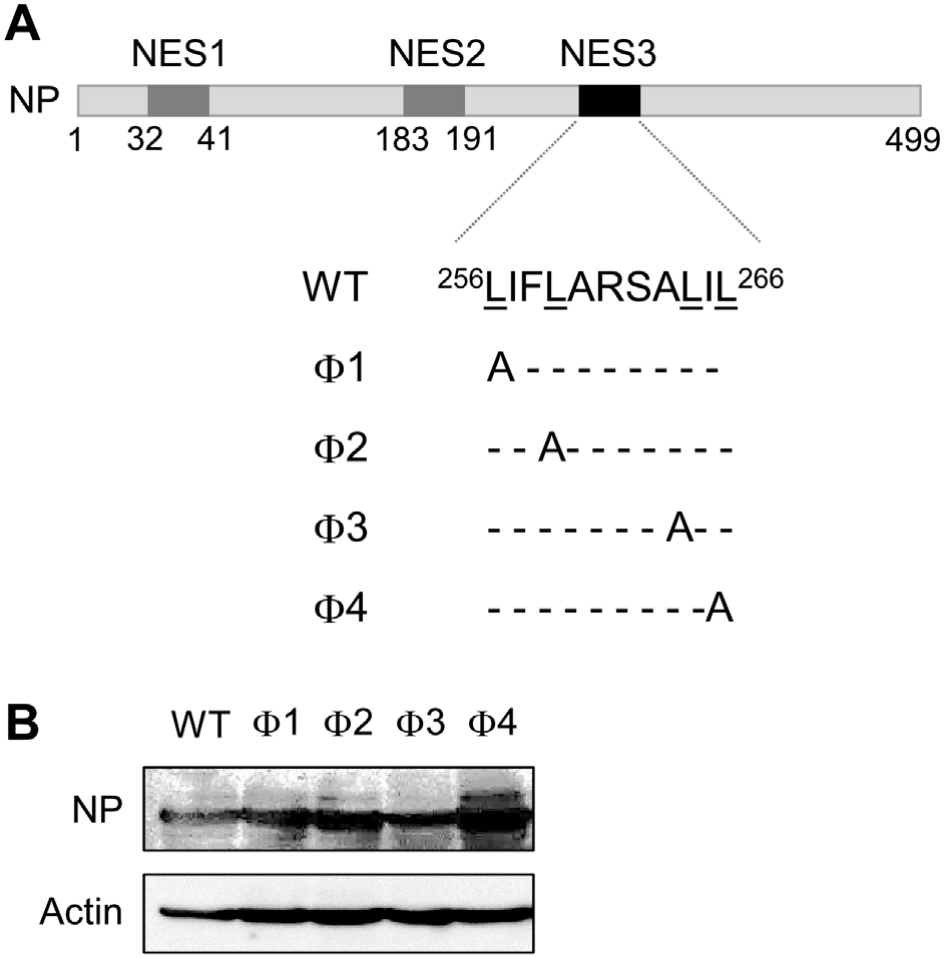
Construction and expression of NP-NES3 consensus sequence mutants. (A) NP-NES harboring mutations in individual or all Φ residues (leucine was replaced by alanine by site-directed mutagenesis). (B) Expression of wild-type NP and NP-NES3 mutants (Φ1, Φ2, Φ3, and Φ4) was compared by Western blotting with an anti-WSN Ab and an anti-actin MAb.

Since replacing the last two Φ residues (Φ3 and Φ4) within the NES3 consensus of NP with alanine severely affected viral production (Fig. 1), we next (and for the first time) identified which Φ residue(s) plays a critical role in NP-NES3 function by constructing mutants harboring mutations in each Φ residue (Fig. 3A). HEK-293T cells were transfected with PA/pCAGGS, PB1/pCAGGS, and PB2/pCAGGS along with the wild-type or the NES3 mutants (Φ1;L256A, Φ2;L259A, Φ3;L264A, or Φ4;L26A) of NP/pCAGGS and NP/pHH21, and the expression of each NP-NES mutant protein was examined. We found that the expression of each NP-NES mutant was comparable with that of the respective wild-type protein (Fig. 3B).

**Figure 4:**
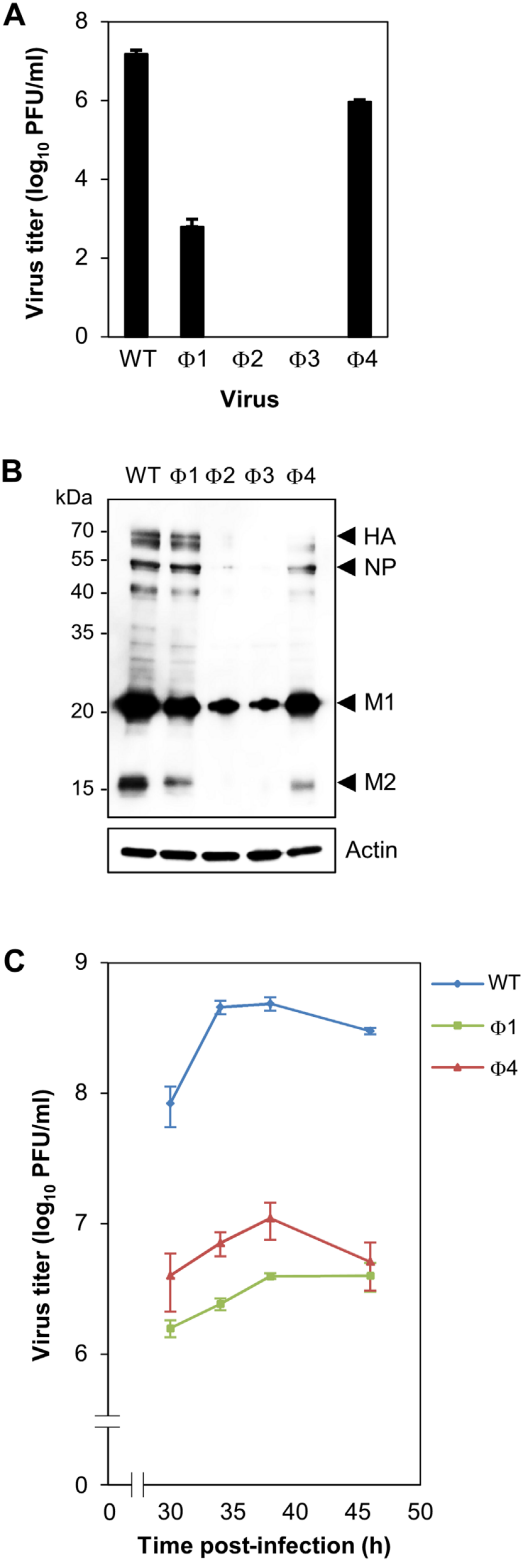
Production, protein expression, and replication kinetics of NP-NES3 mutant viruses. (A) Wild-type and NP-NES3 mutant (Φ1, Φ2, Φ3, and Φ4) viruses were produced by reverse genetics in co-cultures of HEK-293T and MDCK cells. Virus titers in the cell supernatant were calculated in a plaque assay on MDCK cells. Data are expressed as the mean (± SD) PFU/ml from triplicate assays. (B) Comparison of viral protein expression in the producer cells in (A). The cells were collected, lysed, and subjected to Western blotting with an anti-WSN Ab and an anti-actin MAb. The NP, HA, M1, and M2 proteins and molecular weight markers are shown. (C) Replication kinetics were monitored by infecting MDCK cells with equal amounts of each virus (MOI = 0.001). Supernatants containing viruses were collected at the indicated times post-infection and tested in a plaque titration assay. Data are expressed as the mean (± SD) PFU/ml from triplicate titrations.

Infectious virus suspensions of wild-type and NP-NES3 mutants were produced by reverse genetics and titrated in a plaque assay. No virus titers were detected for the Φ2 and Φ3 mutants, which correlates with the reduction in viral protein expression observed in producer cells (Figs 4A and 4B). The titers of the Φ1 and Φ4 mutant viruses were lower (by 10^4^-fold and 10-fold, respectively) than that of the wild-type, and both showed reduced replication kinetics (10^2^-fold lower titer) with respect to the wild-type when inoculated onto cells at the same concentration (Figs 4A and 4C).

**Figure 5:**
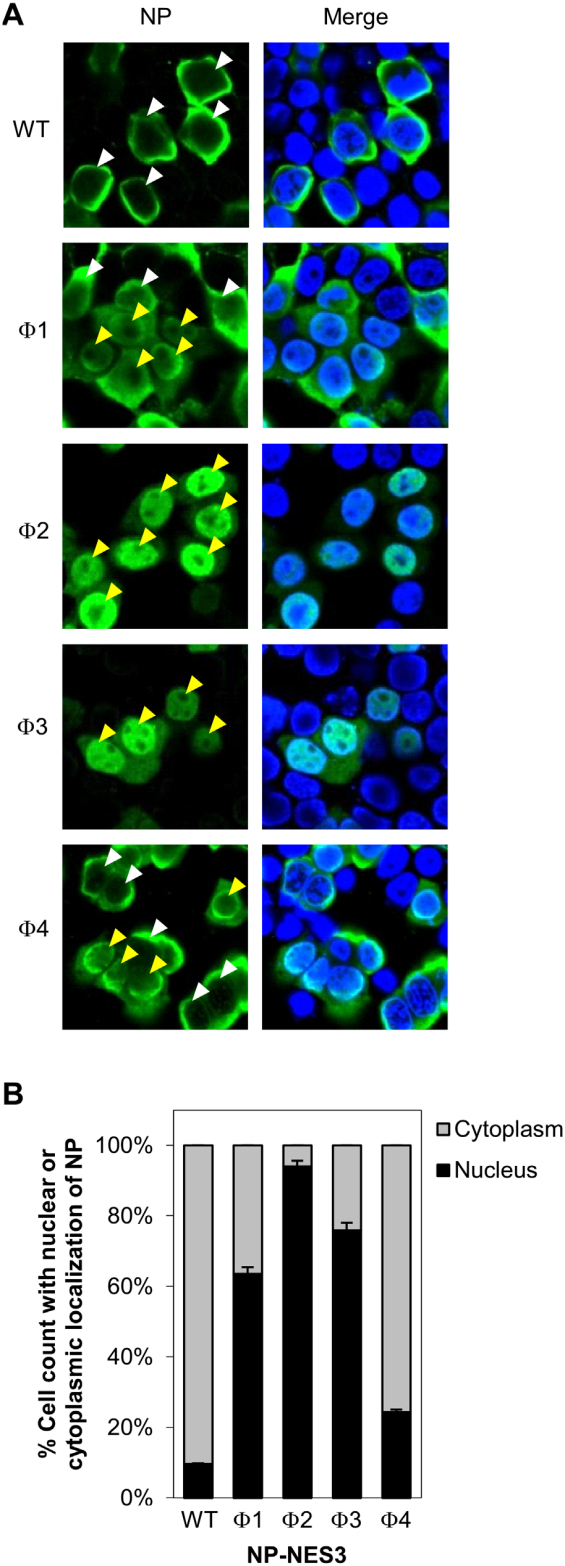
Intracellular localization of NP-NES3 mutant proteins. (A) HeLa cells were grown on cover glass and transfected with pCAGGS encoding wild-type NP-NES3 or its mutants (Φ1, Φ2, Φ3, or Φ4) for 48 h before immunofluorescence staining with an anti-NP MAb followed by anti-mouse Alexa Fluor 488 and Hoechst 333342. The cells were then observed under a confocal laser-scanning microscope. The white and yellow arrow heads indicate predominant localization of NP in the cytoplasm (cytoplasmic staining > nuclear staining) and nucleus (nuclear staining > cytoplasmic staining), respectively. (B) Nuclear localization of NP wild-type and NP-NES3 mutants from A. Data are presented as the percentage (± SD) of total cell count with predominant nuclear or cytoplasmic staining of NP from five separate fields.

**Figure 6:**
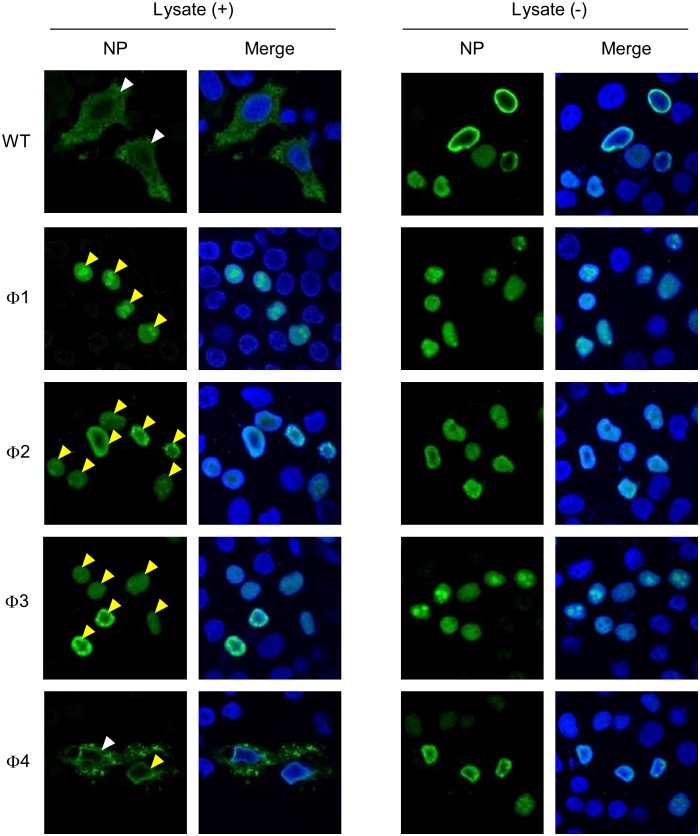
Nuclear export activity of NP-NES3 mutant proteins. HeLa cells growing on cover glass were transfected with pCAGGS encoding wild-type NP-NES3 or its mutants (Φ1, Φ2, Φ3, or Φ4) for 48 h. The cells were then permeabilized with 50 µg/ml digitonin for 5 min on ice. The cytoplasmic components, including NP, were removed by washing (only NP in the nucleus remained). The nuclear export activity of NP was allowed to proceed in the presence of fresh total HeLa cell lysate at 30°C for 1 h (left column). Negative controls were incubated in the absence of total cell lysate (right panel). The cells were then stained with an anti-NP MAb followed by anti-mouse Alexa Flour 488 and Hoechst 333342, and observed under a confocal laser-scanning microscope. The white arrow head indicates nuclear export activity, whereas the yellow arrow head indicates a failure of nuclear export activity.

To examine how NP-NES3 mutants affected viral production and replication, we next examined the subcellular localization of these proteins in HeLa cells transfected with a pCAGGS vector containing wild-type NP or NP-NES3 harboring mutations in Φ1, Φ2, Φ3, or Φ4. Wild-type NP mainly localized in the cytoplasm (90% cell count), although some was present in the nucleus (10% cell count) (Figs 5A and 5B). Transfection with NP-NES3 Φ1, Φ2, or Φ3 mutant caused remarkable increases of the cells with nuclear accumulated NP (64%, 94%, or 76% cell count, respectively) (Figs 5A and 5B). However, localization of Φ4 mutant was almost similar to that of wild-type NP (the Φ4 mutant construction is similar to that reported previously and gave the same result [20]). Next, we performed an *in* *vitro* nuclear export assay using digitonin-permeabilized, semi-intact HeLa cells to examine the nuclear export capacity of each NP-NES3 mutant. After digitonin treatment, the cytoplasmic NP was washed out, meaning that only nuclear NP was examined in the assay. Upon addition of HeLa cell lysate (enriched with various cellular factors, including nuclear export factors, e.g., CRM1), we found that wild-type nuclear NP was exported to the cytoplasm (Fig. 6, left panel); however, there was no change in the amount of nuclear NP in the absence of the cell lysate (Fig. 6, right panel). No nuclear export activity was observed for the NP-NES3 Φ1, Φ2, or Φ3 mutant proteins, as shown by retention of these proteins in the nucleus after addition of the cell lysate (Fig. 6, left panel). By contrast, the NP-NES3 Φ4 mutant protein was partially exported to the cytoplasm in the presence of the cell lysate (Fig. 6, left panel).

Taken together, these data indicate that the nuclear export function of NP, which is necessary for efficient viral production and replication, is dependent on the Φ1, Φ2, and Φ3 residues (and partially dependent on the Φ4 residue) within the NES3 consensus sequence.

### The NP/CRM1 interaction requires Φ2, Φ3, and Φ4 within the NP-NES3 consensus sequence

**Figure 7:**
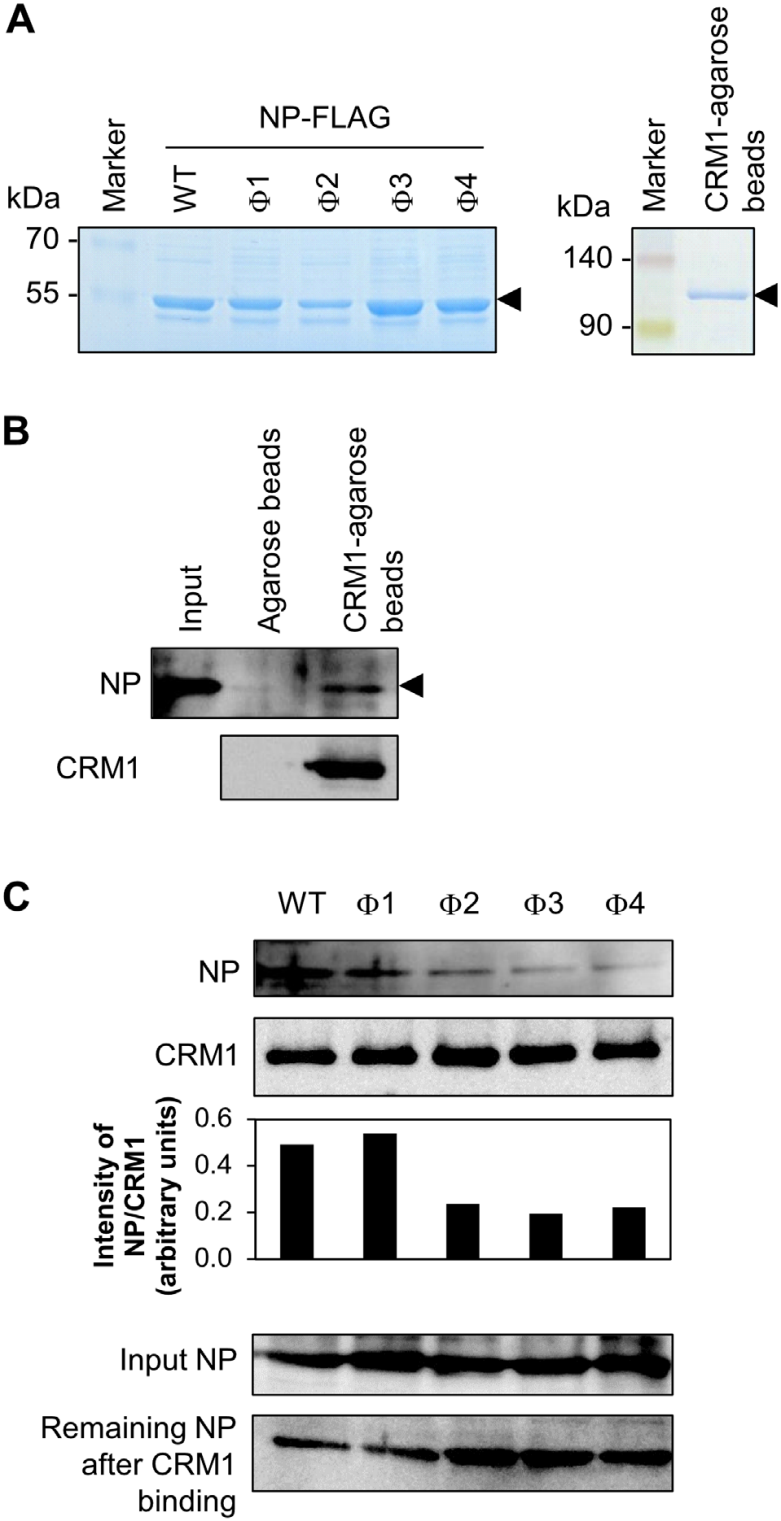
Binding of NP-NES3 mutant proteins to CRM1. (A) FLAG-tagged NP-NES3 wild-type and mutant (Φ1, Φ2, Φ3, and Φ4) proteins were prepared by transfecting the relevant pCAGGS plasmids into HEK-293T cells. The cells were then lysed. The proteins were captured by anti-FLAG agarose beads and eluted with a FLAG-peptide. CRM1-HA agarose beads were prepared by transfecting CRM1-HA/pCAGGS into HEK-293T cells. The cells were then lysed and the proteins were captured on anti-HA agarose beads. The purified proteins and beads were then run in 10% SDS-PAGE gels and stained with Coomassie Brilliant Blue. (B) NP/CRM1 binding was demonstrated by incubating equal amounts of CRM1-HA agarose beads or anti-HA agarose beads alone with purified NP proteins at 4°C for 3 h. After pull-down and washing, the beads were boiled with 4×SDS sample buffer and subjected to 10% SDS-PAGE and Western blot analysis with an anti-WSN Ab and an anti-CRM1 MAb. Input NP at 30% was included. (C) Equal amounts of purified wild-type NP-NES3-FLAG or mutant (Φ1, Φ2, Φ3, and Φ4) protein were co-incubated with CRM1-HA agarose beads at 4°C for 3 h, pulled-down, washed, boiled with 4×SDS sample buffer, and then subjected to Western blot analysis with an anti-WSN Ab and an anti-CRM1 MAb. NP/CRM1 binding affinity was compared by measuring the intensity of the NP band normalized against the CRM1 band. Each 30% input NP protein and remained NP protein after binding with the CRM1-HA agarose beads are also shown.

Several studies show that effective nuclear export of NP requires the CRM1 protein [22], [23]. Treatment with the CRM1 inhibitor, LMB, inhibits nuclear export of an EGFP-NP-NES3 fusion protein, but not that of NP-NES1 or NP-NES2 proteins [20]. No direct interaction between NP-NES3 and CRM1 has been reported; therefore, we next examined which of the Φ residues within the NES3 consensus sequence was necessary for CRM1 binding. Recombinant CRM1-HA was immobilized on anti-HA agarose beads, and wild-type or mutant (NP-NES3 Φ1, Φ2, Φ3, and Φ4) NP-FLAG proteins were purified using anti-FLAG agarose beads followed by FLAG-peptide elusion (Fig. 7A). NP/CRM1 binding was confirmed by pull-down of NP-FLAG proteins with CRM1-tagged agarose beads or anti-HA agarose beads. The results showed that NP specifically bound to the CRM1 protein (Fig. 7B). Subsequently, co-incubation of NP-NES3 mutants with CRM1 showed that the Φ2, Φ3, and Φ4 mutants partially lost their binding affinity for CRM1 (Fig. 7C). This result was consistent with those of previous experiments demonstrating that the Φ2 and Φ3 mutants (but not the Φ4 mutant) showed low nuclear export activity and defective viral production (Figs 4–6). The Φ1 mutant retained its affinity for CRM1 (Fig. 7C), although it showed reduced nuclear export activity and viral replication (Figs 4–6).

These data, together with those presented in Table 3, suggest that Φ2 and Φ3 are critical for viral production and replication by facilitating nuclear export of NP via the CRM1-dependent pathway.

**Table 3:** Summary of viral replication kinetics, nuclear localization, nuclear export capacity, and CRM1 binding of the NP-NES3 consensus sequence mutants.

NP-NES3	Replicationkinetics (%WT)^a^	Nuclear localization(%cell count)^c^	Nuclear exportcapacity^d^	CRM1binding (%WT)^e^
WT	100.0±5.7	9.7±0.4	+	100.0
Φ1 (L256)	1.3±0.3	63.6±4.1	−	109.5
Φ2 (L259)	−b	94.0±3.6	−	48.2
Φ3 (L264)	−b	76.0±4.6	−	39.5
Φ4 (L266)	1.7±0.7	24.4±1.5	±	45.0

a % virus titer ± SD compared with WT at the 46 h of replication kinetic assay from the Fig. 4C.

b No viral rescue by reverse genetics.

c % cell count ± SD with nuclear localization of NP from the Fig. 5B.

d −, ±, + indicate not occur, partially occur, occur of nuclear export capacity, respectively derived from the Fig. 6.

e % intensity of the pull-downed NP band compared with WT from the Fig. 7C.

### Hydrophobic residues within the NP-NES3 consensus sequence are highly conserved among influenza A viruses

Conservation of a domain within any protein suggests that it has an important biological function that has been maintained throughout evolution. Therefore, we examined the conservation ratio of each Φ residue within the NP-NES3 protein of human, avian, and swine influenza A viruses. Full-length NP sequences were collected from the NCBI Influenza Virus Resource (http://www.ncbi.nlm.nih.gov/genomes/FLU/FLU.html) and analyzed for the conservation of Φ1 (L256), Φ2 (L259), Φ3 (L264), and Φ4 (L266) using Perl scripts. We found that all the Φ residues within the NP-NES3 consensus sequence were highly conserved; in particular, there were no mutations in the Φ3 residue within swine influenza NP sequences (Table 4). This result indicates that the NP-NES-3 consensus sequence has been evolutionally maintained to play a significant biological function in host cells.

**Table 4:** Conservation of hydrophobic (Φ) residues within the NP-NES3 consensus sequences of influenza A viruses.

NP-NES3	Human		Avian		Swine		All	
	(n = 1,549)		(n = 2,752)		(n = 931)		(n = 5,232)	
	Residue	%	Residue	%	Residue	%	Residue	%
Φ1	L	99.61265	L	99.85369	L	99.67672	L	99.75053
Φ2	L	92.89864	L	99.56108	L	93.21121	L	96.44982
Φ3	L	99.93544	L	99.89027	L	100	L	99.92324
Φ4	L	99.93544	L	99.78054	L	99.78448	L	99.82729

## Discussion

Recently, several novel influenza A viral NES consensus sequences have been identified [15], [19], [20]. The presence of these NES sequences, together with NLS consensus sequences in many viral proteins [24], [25], indicates that the nuclear-cytoplasmic shuttling of viral proteins is important for the viral life cycle. Here, we examined the roles of seven NES consensus sequences (M1-NES, NS1-NES, NS2-NES1, NS2-NES2, NP-NES1, NP-NES2, and NP-NES3) in viral production, replication, nuclear export, and CRM1 binding. The results suggest that the NP-NES3 consensus sequence is the most important. Indeed, no NP-NES3 mutant (L264A/L266A) viruses were rescued using reverse genetics, and the mutant NP-NES3 protein was retained in the host cell nucleus. Evidence suggests that NP, the major subunit of vRNP, promotes vRNP nuclear export by interacting with CRM1 [22]. This may have been the reason for the failure to rescue mutant NP-NES3 viruses. However, the localization of the NP-NES1 and NP-NES2 mutants did not change, and they had only a partial effect on replication kinetics, which may suggest an as-yet unidentified role for these NES consensus sequences in viral replication. Indeed, the nuclear export function of NP is thought to involve viral protein expression at the post-transcriptional level, and a previous study identified a correlation between NP nuclear export and NP oligomerization [20]. Here, we used NP-NES3 harboring mutations in the Φ3 and Φ4 (L264A/L266A) residues, since Φ3 and Φ4 within the NES consensus sequence are crucial for NES function [8]. It is noteworthy that this mutant is different from that examined in a previous study, which harbored a mutation in Φ4 (L266A) only [20]. Thus, the present study is the first to show the effects of individual NP-NES mutants (NP-NES1, NP-NES2, and NP-NES3) on viral rescue and replication kinetics. The results also demonstrate that the NS2-NES1 (M19T/L21S), NS2-NES2 (L38A/L40H), NS1-NES (L144A/L146A), and M1-NES (L66A/V68A) mutants were retained in the nucleus and exhibited reduced replication kinetics. Since NS2 was originally thought to facilitate the export of vRNP from the nucleus [12], defects in NS2-NES may cause nuclear retention and reduced replication kinetics. The ineffective replication of M1-NES mutants may also be explained by the involvement of the M1 protein in vRNP nuclear export [26]. Unfortunately, it is still not understand in-depth why the NP-NES3 consensus sequence is necessary for the influenza A virus life cycle.

Most of the identified NES consensus sequences are located within viral proteins that form components of the vRNP complex (NS2, NP, and M1) [12], [15], [19], [20]. Nuclear export of M1-NES, NP-NES1, and NP-NES2 was CRM1-independent, whereas that of NP-NES3, NS2-NES1, and NS2-NES2 was CRM1-dependent. This may indicate that the virus has evolved more than one mechanism for the nuclear export of vRNP. The results presented herein indicate that other NESs cannot rescue the nuclear export function of NP-NES3. However, it is still unclear whether all of the NESs function in a coordinated manner.

This study also shows that the Φ2 (L259) and Φ3 (L264) residues within the NES3 consensus sequence are critical for the CRM1-binding and nuclear export functions of NP; therefore, they affect viral production and replication. However, the inhibitory effect of the Φ2 and Φ3 mutations in the reverse genetics experiments (failure to rescue viruses) was greater than that of CRM1-binding (which showed only a partial inhibitory effect). Mutation at the Φ4 (L266) residue also affected CRM1-binding and viral replication while partially decreased nuclear export function. By contrast, the Φ1 (L256) mutation reduced nuclear export function and replication kinetics without interfering CRM1-binding. In addition, a previous study also shows that nuclear export of wild-type NP is partially reduced by LMB [20]. Taken together, these data suggest that viral replication depends, at least in part, on the NP/CRM1 interaction, and may indicate the presence of an as-yet-unidentified cellular factor that interacts with NP-NES3 Φ1, Φ2, and Φ3 in parallel with CRM1 to facilitate nuclear export. In addition, the NP/CRM1 interaction may be mediated by an additional unidentified domain of NP. Various host factors are involved in the nuclear-cytoplasmic transport of vRNPs and viral proteins, including nucleporin62 (Nup62), Nup98, Nup153, and E1B-AP5 [27]–[30]. However, we did not detect any interaction between these four cellular factors and the NP protein in our pull-down assay (data not shown). The characteristic sequence of NP-NES3 (^256^ LIFLARSALIL ^266^, Φ residues underlined) is slightly different from that of traditional NESs, e.g., Rev (^73^ LQLPPLERLTL ^83^) and PKI (^35^ LNELALKLAGLDI ^47^) [31]. Therefore, further detailed analyses (e.g., X-ray crystallography and nuclear magnetic resonance spectroscopy) are required if we are to fully understand the interaction between NP and CRM1. This difference may also suggest that NP-NES3 is able to interact with more than one cellular protein partner.

Since both the NP and HIV-1 Rev proteins comprise the RNA binding domain and the NES consensus sequence [20], [31]–[33], it may be that the role of NP during viral RNA nuclear export is similar to that of Rev. Although the nuclear export of influenza viral mRNAs is mainly dependent on the cellular NXF1/TAP pathway [34], [35], each viral mRNA species depends upon NXF1/TAP to a different extent; in particular, the nuclear export of PB2 and NP mRNAs is partially depend on NXF1/TAP [36]. Since nuclear export of influenza viral mRNA is not dependent upon the CRM1 pathway [29], [34], [35], NP may also interact with an as-yet-unidentified cellular factor to support NXF1/TAP during the nuclear export of viral mRNA. Further identification of the novel host factor(s) involved in NP nuclear export function is required to facilitate a better understanding of the influenza virus life cycle.

In summary, the present study shows for the first time that the most important influenza viral NES consensus sequence is NP-NES3, and that its export functions are dependent upon the Φ2 and Φ3 residues within the NES. Viral replication is dependent upon the nuclear export function of NP, but also partially upon the NP/CRM1 interaction. The Φ residues within the NES are highly conserved among influenza A virus species. The currently available antiviral drugs, which are mainly NA inhibitors (oseltamivir and zanamivir) or M2 channel blockers (amantadine and rimantadine), have problems in terms of safety and/or the emergence of viral resistance [37]. Thus, the NP-NES3 consensus sequence may be a potential target for the development of novel antiviral drugs.

## Materials and Methods

### Cells, viruses, and antibodies

HEK-293T, MDCK, and human cervical HeLa cells were maintained in Dulbecco’s Modified Eagle’s Medium (DMEM; Gibco) supplemented with 10% fetal bovine serum (FBS; Gibco) at 37°C/5% CO_2_. Infectious wild-type and NES mutant viruses (produced by reverse genetics, as described below) were derived from influenza A/WSN/33. A polyclonal antibody (Ab) against influenza A/WSN/33 virus proteins (anti-WSN Ab: a kind gift from Dr. Kazufumi Shimizu, Nihon University School of Medicine) was used for Western blot analysis together with horseradish-peroxidase (HRP)-conjugated goat anti-rabbit IgG (Amersham Bioscience). Monoclonal antibodies (MAbs) against M1 (Santa Cruz), NS1 (Santa Cruz), CRM1 (BD Biosciences), and β-actin (Sigma), and an HRP-conjugated goat anti-mouse IgG (Amersham Bioscience), were also used. Anti-NS1 MAb (Santa Cruz), anti-NS2 Ab (GeneTex), anti-NP MAb (Santa Cruz), anti-M1 MAb (Abcam), Alexa Fluor 488 rabbit anti-mouse IgG (Invitrogen), and Alexa Fluor 488 goat anti-rabbit IgG (Invitrogen) were used for immunofluorescence staining.

### Plasmid construction

The following primers and templates (influenza A/WSN/33) were used to construct the NES mutants: M1-NES (forward: 5′-TTCACGGCCACCGCGCCCAGTGAGCGGGGACT-3′, and reverse: 5′-CTGGGCGCGGTGGCCGTGAACACAAATCCTAA-3′; M1/pHH21); NS1-NES (forward: 5′-GAGACTCTAATATTACTAAGGGCCTTCACCGA-3′, and reverse: 5′-CTTAGTAATATTAGAGTCTCCAGCCGGTCAAA-3′; NS/pHH21); NS2-NES1 (forward: 5′-ACGCAGTCGGGGTCCTCATCGGAGGA-3′, and reverse: 5′-CCGACTGCGTTTTTGACATCCTCATCA-3′; NS/pHH21); and NS2-NES2 (forward: 5′-CGAGTCAGCGAAACACTACAGAGATTCGCTTGG-3′, and reverse: 5′-CTGTAGTGTTTCGCTGACTCGAACTGTGTTATT-3′; NS/pHH21). For the NP-NES mutations, NP/pHH21 and NP/pCAGGS were used as templates along with the following primers: NP-NES1 (forward: 5′-GGACGAGCCTACGCCCAAATGTGCACCGAACT-3′, and reverse: 5′-ATTTGGGCGTAGGCTCGTCCAATTCCACCAAT-3′); NP-NES2 (forward: 5′-GGAACAGCGGTGGCGGAATTGATCAGAATGAT-3′, and reverse: 5′-AATTCCGCCACCGCTGTTCCAACTCCTTTGAC-3′); NP-NES3 (forward: 5′-TCTGCAGCCATAGCGAGAGGGTCAGT-3′, and reverse: 5′-GCTATGGCTGCAGACCGTGCTAGAAA-3′); NP-NES3 Φ1 (forward: 5′-GAAGATGCCATCTTTCTAGCACGGTC-3′, and reverse: 5′-AAGATGGCATCTTCGAACTCAGCATT-3′); NP-NES3 Φ2 (forward: 5′-ATCTTTGCAGCACGGTCTGCACTCAT-3′, and reverse: 5′-CGTGCTGCAAAGATGAGATCTTCGAA-3′); NP-NES3 Φ3 (forward: 5′-TCTGCAGCCATATTGAGAGGGTCAGT-3′, and reverse: 5′-AATATGGCTGCAGACCGTGCTAGAAA-3′); and NP-NES3 Φ4 (forward: 5′-CTCATAGCGAGAGGGTCAGTTGCTCA-3′, and reverse: 5′-CCTCTCGCTATGAGTGCAGACCGTGC-3′).

NP-FLAG was amplified from the NP/pCAGGS plasmid using forward primer 5′-AAACTCGAGATGGCGACCAAAGGCACCAA-3′ and reverse primer 5′-AAAGCGGCCGCTTACTTGTCATCGTCGTCCTTGTAATCTTAATTGTCGTACTCCTCT-3′ (*XhoI* and *NotI* sites underlined), while CRM1-HA was generated from HeLa cell cDNA using forward primer 5′-AAACTCGAGATGCCAGCAATTATGACAATG-3′ and reverse primer 5′-AAAAGCGGCCGCTTAAGCGTAATCTGGAACATCGTATGGGTAATCACACATTTCTTCTGG-3′ (*XhoI* and *NotI* sites underlined). Both PCR products were cut with *Xho*I/*No*tI and cloned into the modified pCAGGS plasmid [38]. The Flag-NP-NES3 Φ1, Φ2, Φ3, and Φ4 mutants were constructed using the same primer sets used to construct the untagged NP-NES3. All plasmids were verified by DNA sequencing using the ABI PRISM BigDye Terminator Cycle Sequencing Ready Reaction Kit (Applied Biosystems).

### Virus production and viral protein expression

Infectious viral particles were produced by reverse genetics, as previously described [5], [21] with some modifications. Briefly, HEK-293T (4×10^5^ cells for efficient transfection) and MDCK (6×10^5^ cells for viral amplification) cells were co-cultured overnight in 6-well plates. Viral genomic plasmids (PA/pHH21, PB1/pHH21, PB2/pHH21, NP/pHH21, M/pHH21, NS/pHH21, HA/pHH21, and NA/pHH21) and protein expression plasmids (PA/pCAGGS, PB1/pCAGGS, PB2/pCAGGS, and NP/pCAGGS) [1 µg of each (0.5 µg for PA/pCAGGS)] were mixed in Opti-MEM (Invitrogen) and transfected into cells for 6 h using FuGene HD (Promega). The medium was then replaced with fresh DMEM (without FBS) supplemented with 1 µg/ml of tosylsulfonyl phenylalanyl chloromethyl ketone-treated trypsin (Trypsin-TPCK, Worthington Biochemical Corporation) and cultured for a further 72 h. Supernatants containing viruses were then collected, clarified by centrifugation, and stored at –80°C until use. The viral producer cells were washed with phosphate-buffered saline (PBS), collected, lysed with NET buffer (10 mM Tris-Cl (pH 7.4), 150 mM NaCl, 1 mM EDTA, 1% NP-40) supplemented with protease inhibitor cocktail (Roche), boiled with 4×sodium dodecyl sulfate (SDS) sample buffer (40% glycerol, 240 mM Tris-Cl (pH 6.8), 8% SDS, 0.04% bromophenol blue, and 5% β-mercaptoethanol), and subjected to 10% SDS-polyacrylamide gel electrophoresis (PAGE) followed by Western blotting with an anti-WSN Ab.

Expression of NP-NES mutant proteins was compared by co-transfecting PA/pCAGGS, PB1/pCAGGS, PB2/pCAGGS, and wild-type or NES mutants of NP/pCAGGS and NP/pHH21 (i.e., NP-NES1, NP-NES2, NP-NES3, NP-NES3 Φ1, NP-NES3 Φ2, NP-NES3 Φ3, or NP-NES3 Φ4) into HEK-293T cells (1.2×10^5^) using FuGene HD. The cells were lysed with NET buffer and subjected to Western blotting as described above.

### Virus titration

Viral titers were determined in a plaque assay as described previously [5], [39]. Briefly, MDCK cells (5×10^5^) were grown overnight in 6-well plates. Next, the cells were washed with PBS, infected with viral suspensions (serially diluted 1∶10), and incubated for 1 h to allow viral absorption. Excess virus was removed by washing with PBS. Next, 1% agarose (Lonza) in DMEM (without FBS) supplemented with 1 µg/ml trypsin-TPCK was added to each well. After the agarose had solidified, the plate was flipped and incubated in a CO_2_ incubator for 48 h. The agarose was removed and the cells were stained with crystal violet before plaque counting. The number of plaque forming units (PFU)/ml was then calculated.

### Assay of viral replication kinetics

MDCK cells (5×10^5^) were infected with each virus at an MOI of 0.001 and grown in 6-well plates, as described previously [5]. The cells were allowed to absorb virus for 1 h in a CO_2_ incubator before being extensively washed with PBS. The medium was replaced with DMEM (without FBS) supplemented with 1 µg/ml trypsin. At the indicated time-points post-infection, the virus-containing supernatants were collected and titrated in a plaque assay.

### Immunofluorescence microscopy

HeLa cells (1×10^5^) were seeded on coverslips in 12-well plates and then transfected for 48 h with pCAGGS encoding wild-type or mutant NESs from NS2 or NP using FuGene HD. Immunofluorescence staining was performed by fixing the cells with 4% paraformaldehyde for 10 min at room temperature. The paraformaldehyde was then replaced with cold methanol and the cells maintained at –20°C for 20 min. The cells were then washed with PBS and incubated with anti-NS2 or anti-NP Abs for 1 h at room temperature. After further washing with PBS, Alexa Fluor 488 (1∶200) was added for 1 h at room temperature in the dark. Nuclei were stained with Hoechst 33342 (1∶4000; ImmunoChemistry Technologies LLC) for 5 min in the dark. The coverslips were then rinsed with PBS and mounted on glass slides. Fluorescence images were obtained under a confocal laser-scanning microscope (FV 1000, Olympus). For the NP-NES3 Φ1, Φ2, Φ3, and Φ4 mutants, the number of cells showing predominant nuclear (nuclear staining > cytoplasmic staining) or cytoplasmic (cytoplasmic staining > nuclear staining) localization of NP was counted in five separate fields, the percentages ± SD of each cell population was calculated plotted.

### *In vitro* nuclear export assay

HeLa cells (1×10^5^) were cultured on coverslips in 12-well plates, transfected with pCAGGS harboring wild-type NP or mutant NP-NES3 Φ1, Φ2, Φ3, or Φ4, and then examined in a nuclear export assay [5]. Briefly, the cell membrane was selectively permeabilized by treatment with 50 µg/ml digitonin (Sigma) for 5 min on ice. The cells were then incubated with apyrase (10 units/ml; Sigma) on ice for 5 min to deplete intracellular ATP, washed, and then incubated in PBS to remove all cytoplasmic components. Fresh HeLa cell lysates (prepared by lysing cells in 10 mM Tris-Cl (pH 7.4), 2 mM MgCl_2_, 3 mM CaCl_2_, and 0.3 M sucrose) supplemented with protease inhibitor cocktail, 5 mg/ml bovine serum albumin, 1 mM ATP (Sigma), 5 mM creatine phosphate (Roche), and 32 units/ml creatine kinase (Sigma) were added to the cells and incubated at 30°C for 1 h to allow nuclear export to proceed. The cells were then stained with an anti-NP Ab followed by anti-mouse Alexa Fluor 488 and Hoechst 33342. Fluorescence images were taken under a confocal laser-scanning microscope (FV 1000, Olympus).

### Affinity binding assay

NP-FLAG (wild-type or NES mutant) proteins and CRM1-HA (conjugated to the anti-HA agarose beads) were prepared from transfected HEK-293T cells [5], [38]. Briefly, HEK-293T were transfected with pCAGGS encoding NP-FLAG (wild-type NP or mutant NP-NES3 Φ1, Φ2, Φ3, or Φ4) or CRM1-HA, and then lysed with NET buffer supplemented with protease inhibitor cocktail for 1 h at 4°C. The total cell lysate was clarified by centrifugation and then incubated with anti-FLAG (Sigma) or anti-HA (Sigma) antibodies conjugated to agarose beads at 4°C for 3 h on a rotor. The CRM1-HA agarose beads were washed with NET buffer and kept at 4°C until required. The NP-FLAG proteins were eluted from anti-FLAG agarose beads by co-incubation with 100 µg/ml FLAG-peptide (Sigma) at 4°C for 3 h. The purified CRM1-HA agarose beads and NP proteins were run on 10% SDS-PAGE gels and subsequently stained with Coomassie Brilliant Blue (Mixell). The binding assay was performed by co-incubating 10 µl of CRM1-HA agarose beads (50% slurry) with 10 µg of NP-FLAG proteins in TB buffer (20 mM Hepes pH 7.4, 110 mM potassium acetate, 2 mM magnesium acetate, and 1 mM EGTA in a total volume of 500 µl supplemented with protease inhibitor cocktail) for 2 h at 4°C, in a 1.5 ml tube, on a rotor. The agarose beads were washed ten times with TB buffer (beads were spun down at 3,000×g for 1 min between washes), boiled with 4×SDS sample buffer, and then subjected to Western blotting with an anti-WSN Ab and an anti-CRM1 MAb. The intensity of each NP band was measured using FluorChem software (Alpha Innotech) and normalized to the intensity of CRM1 before plotting on a graph.

### Western blot analysis

Each protein sample was boiled in 4×SDS sample buffer and electrophoresed in 10% SDS-PAGE at 30 mA for 1 h. Proteins were then transferred to a polyvinylidene difluoride membrane (Millipore) using a Trans-Blot Turbo apparatus (Bio-Rad). The membrane was blocked in 5% skimmed milk (in PBST [PBS/0.1% Tween-20]) for 1 h, incubated with the primary antibody overnight at 4°C, washed with PBST, and then incubated with the appropriate secondary antibody at room temperature for 1 h. The chemiluminescent signal was developed using SuperSignal West Pico chemiluminescent substrate (Thermo Scientific) and imaged using a FluorChem 5500 (Alpha Innotech).

### Calculation of the conservation ratio

NP protein sequences from human, avian, and swine influenza viruses were downloaded from NCBI’s Influenza Virus Resource [40] and filtered using the following parameters: type A, full-length only, and whole collection date. Each group of NP sequences was aligned by MAFFT software (version 7.127 b) using the FFT-NS-2 method [41]. After removing defective sequences, the remaining sequences (human, 1,549; avian, 2,752; and swine, 931) were used to calculate the conservation ratios of NP-NES Φ1, Φ2, Φ3, and Φ4.
